# The thermodynamic basis of glucose‐stimulated insulin release: a model of the core mechanism

**DOI:** 10.14814/phy2.13327

**Published:** 2017-06-30

**Authors:** David F. Wilson, Abigail T. J. Cember, Franz M. Matschinsky

**Affiliations:** ^1^ Department of Biochemistry and BiophysicsPerelman School of MedicineUniversity of PennsylvaniaPhiladelphiaPennsylvania

**Keywords:** Diabetes, glucokinase, glucose homeostasis, glucose sensing, insulin release, oxidative phosphorylation, pancreas

## Abstract

A model for glucose sensing by pancreatic *β*‐cells is developed and compared with the available experimental data. The model brings together mathematical representations for the activities of the glucose sensor, glucokinase, and oxidative phosphorylation. Glucokinase produces glucose 6‐phosphate (G‐6‐P) in an irreversible reaction that determines glycolytic flux. The primary products of glycolysis are NADH and pyruvate. The NADH is reoxidized and the reducing equivalents transferred to oxidative phosphorylation by the glycerol phosphate shuttle, and some of the pyruvate is oxidized by pyruvate dehydrogenase and enters the citric acid cycle. These reactions are irreversible and result in a glucose concentration–dependent reduction of the intramitochondrial NAD pool. This increases the electrochemical energy coupled to ATP synthesis and thereby the cellular energy state ([ATP]/[ADP][Pi]). ATP and Pi are 10–100 times greater than ADP, so the increase in energy state is primarily through decrease in ADP. The decrease in ADP is considered responsible for altering ion channel conductance and releasing insulin. Applied to the reported glucose concentration–dependent release of insulin by perifused islet preparations (Doliba et al. 2012), the model predicts that the dependence of insulin release on ADP is strongly cooperative with a threshold of about 30 *μ*mol/L and a negative Hill coefficient near −5.5. The predicted cellular energy state, ADP, creatine phosphate/creatine ratio, and cytochrome c reduction, including their dependence on glucose concentration, are consistent with experimental data. The ability of the model to predict behavior consistent with experiment is an invaluable resource for understanding glucose sensing and planning experiments.

## Introduction

The pancreatic islets of Langerhans, microscopic clusters composed of insulin, glucagon, and somatostatin‐producing cells, function as the central regulator of the body's fuel metabolism. The *β*‐cells, which contribute as much as 70% of cells and 90% of the endocrine cell mass, are responsible for fuel‐induced release of insulin and have been extensively studied. The studies have focused on glucose‐stimulated insulin biosynthesis and release because insulin has a central role not only in the physiology and pathology of glucose but also in fuel homeostasis. The concept has developed that stimulus secretion coupling in *β*‐cells is primarily due to increased glucose metabolism which changes the adenine nucleotide levels (primarily lowering of free MgADP). Lowering MgADP inhibits potassium efflux, thereby depolarizing the cell membrane and augmenting calcium entry into the cell, and this triggers hormone release. This basic concept of stimulus secretion coupling in chromaffin cells was originally proposed by W.W. Douglas in his 1967 Gaddum lecture (Douglas 1968). In the *β*‐cell signaling pathway, glucokinase (a glucose phosphorylating enzyme) provides the measure of the glucose concentration and the K‐ATP/SUR1 channel complex, which controls the membrane potential, is the target of the metabolic coupling factors ATP^4−^ and MgADP (Matschinsky and Ellerman 1968; Dean and Mathews 1970; Ashcroft et al. 1984). Continuing study of fuel‐induced insulin secretion has led to numerous additional putative coupling factors and coupling mechanisms. These are typically put forward as explanations for specific experimental findings, such as the provocative observation of a marked glucose‐induced augmentation of insulin release from *β*‐cells that are fully depolarized either by high potassium or pharmacologically by K‐ATP channel inhibitors (Henquin et al. 2003). The large number of putative coupling factors makes it difficult to establish which have primary and secondary roles in determining fuel‐stimulated insulin release. This ambiguity is reflected in different and sometimes contradictory opinions expressed in reviews by experts in the field (e.g. Matschinsky and Ellerman 1973; MacDonald 1990; Henquin et al. 2003; Jensen et al. 2008; Prentki et al. 2013). A thermodynamically grounded, mechanistic model of the signaling pathway that quantified the contributions of glycolysis, the citric acid cycle, and oxidative phosphorylation to glucose sensing would be an invaluable asset for understanding stimulus secretion coupling of pancreatic *β*‐cells. This article reports our efforts to develop such a model.

In short, glucose‐stimulated insulin release is a tightly integrated activity that may be thought of as having three parts: the glucose sensing element that couples glucose concentration into oxidative phosphorylation, oxidative phosphorylation itself, and a mechanism for releasing insulin that involves both potassium and calcium channels in an energy state–dependent manner. The glucose sensor of pancreatic *β*‐cells is glucokinase (Meglasson et al. 1983; Garfinkel et al. 1984; Meglasson and Matschinsky 1986; Matschinsky 1990; Froguel et al. 1993; Matschinsky et al. 1993; Doliba et al. 2012), for which the activity is dependent on the concentration of glucose in the blood in the physiological range. Glucokinase uses ATP to convert the glucose into glucose‐6‐phosphate (G‐6P). This reaction is both irreversible and not inhibited by its product, so the G‐6‐P formed must be removed by glycolysis as rapidly as it is synthesized. Glycolysis produces two moles each of NADH, ATP, and pyruvate for each mole of G‐6‐P metabolized. The *β* cells have low levels of lactate dehydrogenase and monocarboxylate transporter (Sekine et al. 1994; Sweet and Matschinsky 1995; Sweet et al. 1996; Schuit et al. 1997; Ishihara et al. 1999; Otonkoski et al. 2003, 2007). The former limits reduction of pyruvate to lactate, while the latter limits export of pyruvate (and lactate) from the cell. Instead, the NADH is reoxidized to NAD^+^ using the glycerol phosphate shuttle (MacDonald 1990; Dukes et al. 1994; Schuit et al. 1997; Eto et al. 1999; Madiraju et al. 2014), and the reducing equivalents are transferred to the mitochondrial respiratory chain at the level of isopotential group that includes the b cytochromes and ubiquinone. The low lactate dehydrogenase and monocarboxylate transporter activities result in a glucose concentration–dependent increase in the concentration of pyruvate, about 20% of which enters the mitochondria, and is oxidized by PDH and then to CO_2_ and water (Matschinsky et al. 1993; Sweet and Matschinsky 1995; Liang 1996; Doliba et al. 2012) through the citric acid cycle.

Several efforts to develop mathematical models (representations) of glucose sensing by the pancreatic *β*‐cells have been published (Garfinkel et al. 1984; Sweet and Matschinsky 1995). Modeling is an attempt to quantify the relationships among the relevant parameters, representing them with mathematical equations which mimic the experimentally observed metabolic behavior. As such, a model is expected to include most, if not all, of the kinetically important parameters, and a successful model predicts behavior that closely approximates that observed experimentally. Successful models contribute substantially to our understanding by providing credence to the mechanisms used to develop the model. Such models also serve as quantitative frameworks that integrate the behavior of the putative regulatory parameters and can be used to conduct virtual experiments with predicted results, thereby helping to optimize experimental design. The published models for glucose sensing have been partially successful. The kinetic behavior of glucokinase is quite well understood and therefore could be effectively modeled, but a critical part of the sensing system, oxidative phosphorylation, had to be represented by ad hoc equations. A mechanism‐based model for oxidative phosphorylation is now available, however, that has been shown to be consistent with energy metabolism in exercising muscle (Marsh et al. 1993; McCann et al. 1995; Haseler et al. 1999; Wilson 2015a, 2016, 2017), as well as in other cells and tissues (Wilson et al. 1979; Wilson and Vinogradov 2014, 2015; Wilson 2015b, 2017). The program for oxidative phosphorylation in MatLab (www.mathworks.com) as well as that for glucose sensing used in the present article can be found at the URL: http://www.med.upenn.edu/biocbiop/faculty/wilson/index.html. We have coupled this model for oxidative phosphorylation, for which the values of the kinetic and thermodynamic parameters are already established, to a mechanism‐based description of the glucokinase/glycolysis part of the sensor system. Predictions of the resulting model are shown to be consistent with the experimental data on glucose sensing obtained using both isolated islets and *β*‐cell lines. This provides substantial credence to the mechanisms used in the model and indicates that the model has substantial predictive power.

## Methods

**Figure 1:**
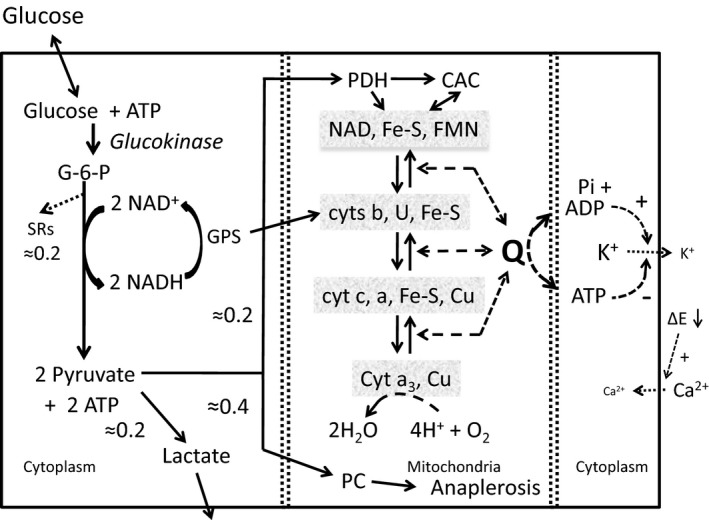
A schematic for glucose sensing by the pancreatic *β*‐cells. The schematic shows the key components of glucose sensing as incorporated into the model, Glucokinase, in an irreversible reaction, synthesizes G‐6‐P at a rate that is dependent on glucose concentration and this drives the flux through glycolysis to pyruvate. Approximately 20% of the pyruvate is reduced to lactate and leaves the cell, 40% is carboxylated by pyruvate carboxylase (PC), and 20% enters the mitochondria and is oxidized to NADH and CO_2_ through PDH and the citric acid cycle. An additional 20% of the carbon from G‐6‐P is lost through a variety of “spillage” reactions (SRs) related to the concentration of intermediates in glycolysis. These are part of the “safety valve” needed to prevent excessive accumulation of metabolites in the cell. The NADH from glycolysis is reoxidized by the glycerol phosphate shuttle (GPS), transferring the reducing equivalents into the cytochrome b region of the respiratory chain. The influx of reducing equivalents from the shuttle and pyruvate oxidation increase the level of reduction of the intramitochondrial NAD pool, increasing the electrochemical energy (Q) coupled to ATP synthesis. The resulting decrease in ADP lowers K^+^ conductance until the cell depolarizes. This opens the Ca^2+^ channels and calcium enters the cell and triggers insulin release. The isopotential groups of the respiratory chain are shown with representative redox components: U, ubiquinone; Fe‐S, iron sulfur proteins; FMN, flavin mononucleotide; cyts b, the two b cytochromes; Cu, the copper proteins of cytochrome c oxidase; a, cytochrome a; cyt a3, cytochrome a_3_.

A schematic showing the essential parts of the glucose sensing system is shown in Figure 1. In order to systematically develop the model and its mathematical representation, each part is described separately and then the parts fused together to form the whole. The model for oxidative phosphorylation has been published (Wilson et al. 1979; Wilson and Vinogradov 2014, 2015) and is used “as is” except for adding the input from PDH and the glycerol phosphate shuttle. No other changes have been made to this part of the program. The glucose sensor, glucokinase, has been extensively studied, and we have used the expression for G‐6‐P production as a function of glucose concentration published by Matschinsky and coworkers (Meglasson and Matschinsky 1986; Sweet and Matschinsky 1995). Coupling of the production of glucose 6‐phosphate to oxidative phosphorylation occurs by two pathways: 1) the reducing equivalents from NADH generated in the cytoplasm by glyceraldehyde‐3‐phosphate dehydrogenase are transferred to the cytochrome b region of the respiratory chain by the glycerol phosphate shuttle. 2) Pyruvate produced by glycolysis is transported into the mitochondria and is oxidized by pyruvate dehydrogenase (PDH) and the citric acid cycle. The coupling of glucokinase activity to oxidative phosphorylation relies on low monocarboxylate transporter activities in the beta cells so that the pyruvate accumulates in the cell. Both the glycerol phosphate shuttle and PDH are irreversible. Their activities are determined by the rate of G‐6‐P production by glucokinase, increasing with increasing rates of G‐6‐P formation. Experimental measurements indicate that from 10% to 30% of the pyruvate from G‐6‐P is oxidized by PDH and enters the CAC, producing the equivalent of 5 NADH for each pyruvate oxidized. Table 1 presents representative rates of glucose consumption, glucose oxidation, and oxygen consumption by perifused islets as measured by Doliba et al. (2012). The measurements are all expressed in *μ*mol/L s^−1^ assuming the volume of an islet is 2 nL (Ricordi et al. 1990). The same units are used throughout this article in order to facilitate comparison of model and experiment. The figures were prepared using the graphics program Origin^®^ (OriginLab.com).

**Table 1:** The metabolic balance of perifused pancreatic islets (taken from Doliba et al. 2012)

Glucose (mmol/L)	NADH from glycolysis	NADH from pyruvate	NADH oxidized (Oxygen × 2)	Total NADH from glucose^a^
0.5?	2.77 *μ*mol/L s^−1^	1.39 *μ*mol/L s^−1^	13.9 *μ*mol/L s^−1^	4.17 *μ*mol/L s^−1^
3	11.1 *μ*mol/L s^−1^	6.25 *μ*mol/L s^−1^	21.7 *μ*mol/L s^−1^	17.4 *μ*mol/L s^−1^
6	11.1 *μ*mol/L s^−1^	13.9 *μ*mol/L s^−1^	36.7 *μ*mol/L s^−1^	25 *μ*mol/L s^−1^
12	25 *μ*mol/L s^−1^	27.8 *μ*mol/L s^−1^	50 *μ*mol/L s^−1^	52.8 *μ*mol/L s^−1^
24	41.7 *μ*mol/L s^−1^	41.7 *μ*mol/L s^−1^	55 *μ*mol/L s^−1^	83.3 *μ*mol/L s^−1^

The data from Doliba et al. (2012) have been converted into common units of *μ*mol/L s^−1^ based on an assumed islet volume of 2 nL.

a The total production of NADH from glucose was calculated as that from glycolysis plus that from the oxidation of pyruvate. At high glucose, 12 mmol/L and above, the glucose provides NADH in excess of that required for oxygen reduction.

Occam's razor has been applied to the model, i.e. it uses the minimum number of reactions required to describe the sensor system, and the minimum number of parameters for which independent measurements are not available. The use of the minimum number of reactions needed for consistency with experimental observation and the modular design make this a “core” model to which additional parameters can be grafted on as required. The added parameters, however, should not alter the core. Modulators of glucokinase activity (genetic alterations in Km and Vm as well as activators and inhibitors) can be added by appropriate modification of the rate expression for the glucokinase reaction. The final model is consistent with glucose sensing by pancreatic *β*‐cells, from blood glucose to cellular energy state.

## Results

### Glucokinase and sensing of glucose concentration

**Figure 2:**
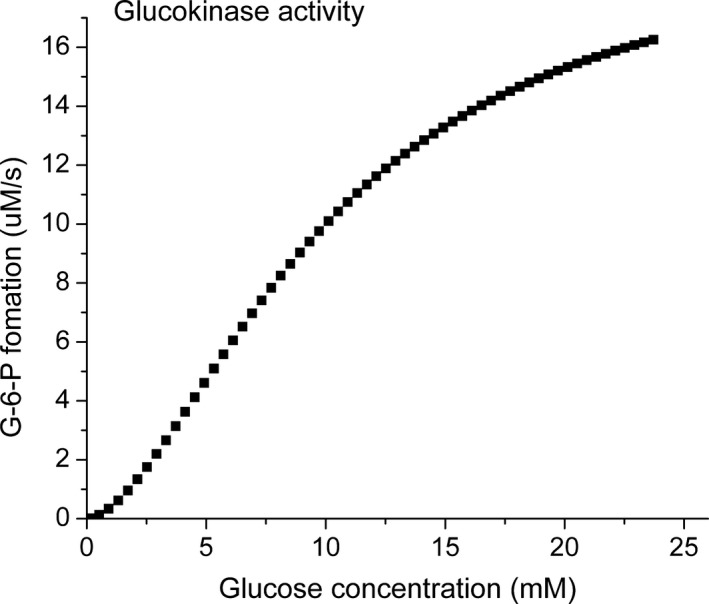
Dependence of the rate of G‐6‐P production by glucokinase on glucose concentration. In the model, the activity of glucokinase is represented by equation (1) using the experimentally determined kinetic equation and parameters: Vm, 17 *μ*mol/L G‐6‐P s^−1^; glucose concentration for half maximal rate, 10 mM; Hill coefficient (n), 1.7.

Glucokinase is responsible for detecting glucose in the environment of the *β*‐cells (Meglasson et al. 1983; Garfinkel et al. 1984; Meglasson and Matschinsky 1986; Matschinsky 1990; Ghosh et al. 1991; Froguel et al. 1993; Matschinsky et al. 1993; Sweet et al. 1996; Doliba et al. 2012). Transport of glucose into the cells is not limiting, and glucokinase produces G‐6‐P at a rate that is an accurate measure of the extracellular concentration. The rate of the glucokinase reaction has been shown to fit the expression for a cooperative enzyme with the glucose concentration dependence for half maximal rate (S_1/2_) of 10 mmol/L and a Hill number of 1.7 (Sweet and Matschinsky 1995). The Km for ATP is about 0.3 mmol/L, well below the physiological concentration of 3–5 mmol/L, and glucokinase is always near saturation with ATP. Near saturation of the binding site on glucokinase, combined with the observation that there is little change in ATP under physiological conditions (Ghosh et al. 1991; Wroblewski et al. 1994; Doliba et al. 2003), means that it is not necessary to include ATP as a variable in calculating glucokinase activity. For the model, the glucose dependence is represented by the expression (Sweet and Matschinsky 1995):

(1) $$V_{G}=V_{m}\times {\left[G\right]}^{1.7}/\left(\text{Kg}+{\left[G\right]}^{1.7}\right)$$

where v_G_ is the rate of formation of G‐6‐P, [G] is the concentration of glucose, and Kg is 3.98*10^−4^ mol/L (0.01^1.7^ = 3.98 × 10^−4^) and that sets the concentration of glucose for half maximal rate of the reaction to 10 mmol/L. The maximal rate of glucokinase (Vm) is reported to be 0.7 *μ*mole glucose/g/min (Sweet and Matschinsky 1995) or about 16.7 *μ*mol/L s^−1^ based on the assumption that there is 0.7 mL water per gram wet weight of islet. The resulting dependence of the rate of G‐6‐P formation as a function of glucose concentration is shown in Figure 2. The glucokinase reaction is irreversible and the amount of G‐6‐P that can be hydrolyzed by G‐6‐Pase, stored as glycogen and/or metabolized through the pentose phosphate pathway is insignificant compared to the glucokinase activity. As a result, further metabolism of G‐6‐P is through glycolysis, and in the steady state, the flux through glycolysis is equal to the rate of production of G‐6‐P.

### Coupling glucokinase activity to oxidative phosphorylation: A. The glycerol phosphate shuttle

As described above, G‐6‐P produced by glucokinase is metabolized through glycolysis. Two moles of pyruvate and two moles of NADH are formed per G‐6‐P, so the maximal rate of formation of each is 33.4 *μ*mol/L s^−1^. The NADH formed in the cytoplasm by glyceraldehyde‐3‐P dehydrogenase has to be recycled to NAD^+^. In *β*‐cells, reoxidation of the cytoplasmic NADH occurs primarily through the glycerol phosphate shuttle, with only about 20% used to reduce pyruvate to lactate which leaves the cells (Matschinsky and Ellerman 1973). Reducing equivalents from the glycerolphosphate shuttle enter the mitochondrial respiratory chain at the level of cytochrome b. This is an irreversible reaction because site 1 of oxidative phosphorylation is bypassed and only 2 ATP are synthesized by oxidative phosphorylation for each NADH oxidized. Although glycolysis and the glycerol phosphate shuttle involve a number of individual reactions, the rate of the glycerol phosphate shuttle is determined by the rate of synthesis of G‐6‐P. Expressed as reducing equivalents (e^−^) s^−1^ (v_S_), this is four times the rate of formation of G‐6‐P by glucokinase (twice the rate of NADH oxidation):

(2) $$V_{S}=4\times V_{G}e^{-}s^{-1}$$

### Coupling glucokinase activity to oxidative phosphorylation: B. Input through pyruvate dehydrogenase

Due to the low lactate dehydrogenase and monocarboxylate transporter activity in *β*‐cells, the concentration of pyruvate in the cytoplasm increases with increase in production of G‐6‐P. As the concentration increases, pyruvate is transported into the mitochondria, where it is both the substrate for pyruvate dehydrogenase (PDH) and an activator for PDH phosphatase (McCormack et al. 1990; Holness and Sugden 2003). The phosphatase activates the dehydrogenase, assuring a pyruvate concentration–dependent flux into the citric acid cycle. Experimentally, 10–30% of the pyruvate is oxidized through PDH and the citric acid cycle, and the contribution to oxidative phosphorylation is equivalent to 5 NADH for each pyruvate oxidized. There are 4 NADH produced by 4 NAD linked dehydrogenases. Succinate dehydrogenase is an FAD‐linked enzyme, but this contributes 2 reducing equivalents and, when the GTP formed from succinyl‐CoA is added to the 2 ATP formed by oxidative phosphorylation, 3 ATP are produced for each FADH_2_ oxidized. This is equivalent to oxidation of an NADH. It has been reported (Sweet and Matschinsky 1995) that the fraction of the pyruvate oxidized is not very, if at all, dependent on the glucose concentration. For the model, it is assumed that 20% of the pyruvate is oxidized. The equivalent of 5 NADH is formed for each pyruvate, and 2 pyruvate are formed per G‐6‐P oxidized. Only 20% of the pyruvate is oxidized (*f* = 0.2); however, the input of reducing equivalents from pyruvate oxidation is approximately equal to that from the glycerol phosphate shuttle. The effect on reduction of the intramitochondrial NAD pool is the sum of the inputs into the cytochrome b region of the respiratory chain, which decreases the rate of NADH oxidation, and that from pyruvate oxidation, which increases the rate of reduction of NAD^+^:

(3) $$V_{B}=4\times 5\times f\times V_{G}e^{-}s^{-1}=2\times V_{S}M\;s^{-1}$$

The value of f is experimentally determined, and not predicted by the model.

### Integration of the input from glucose into oxidative phosphorylation

The model for oxidative phosphorylation (Wilson and Vinogradov 2014, 2015; Wilson 2017) was constructed by adding the expression for near equilibrium from NADH to cytochrome c (Hassinen and Hiltunen 1975; Erecińska et al. 1978; Wilson et al. 1979; Forman and Wilson 1982; Rumsey and Wilson 1996; Wilson and Vinogradov 2014, 2015):

(4) $$\text{NADH}+2c^{3+}\overset{2Q}{⟷}{\text{NAD}}^{+}+H^{+}+2c^{2+}$$

to a mechanistic model for cytochrome c oxidase. Q represents the Gibbs‐free energy in the electrochemical reaction, typically expressed as the potential difference between the redox couples in volts. In order to accommodate the input from glucose, this has been divided into two parts, each representing one of the two coupling sites. Both sites are near equilibrium and the input from glucokinase activity added:

(5) $$b^{2+}+c^{3+}\overset{Q}{⟷}b^{3+}+c^{2+}$$

$$\text{addition}:GK+b^{3+}⟶b^{2+}\;\;\text{rate}=V_{S}$$

and

(6) $$\text{NADH}+2b^{3+}\overset{Q}{⟷}{\text{NAD}}^{+}+H^{+}+2b^{2+}$$

$$\text{addition}:GK+{\text{NAD}}^{+}+H^{+}⟶\text{NADH}\;\;\text{rate}=V_{B}$$

For reactions near equilibrium, the ratio of the forward (v_F_) and reverse (v_R_) rates is directly related to the change in Gibbs‐free energy:

(7) $$\Delta G=-2.303\;\text{RT}\;\text{log}(V_{F}/V_{R})$$

where 2.303 converts the logarithm to the base 10 to the natural logarithm (base e), R is the gas constant, and T is the absolute temperature. At equilibrium, the ratio is 1.0, but in metabolism, there is almost always a net flux in either the forward or reverse direction and the ratio is not exactly 1.0. For the initial conditions, in the absence of glucose, the rate of reduction of oxygen should be subtracted from the reverse rate, making the ratio > 1 and the value of ΔG negative. Although equation 7 is generally applicable, it is important to remember that it is the ratio that is related to ΔG not the forward or reverse fluxes *per se*. However, as long as there are no changes in reaction mechanism or environmental factors that activate or inhibit the reaction and the reactions are near equilibrium (v_F_/v_R_ values between 0.2 and 5) changes in the forward and/or reverse rates have predictable effects on ΔG. These criteria are met for the first two coupling sites of oxidative phosphorylation which are freely reversible (Hassinen and Hiltunen 1975; Erecińska et al. 1978; Wilson et al. 1979; Forman and Wilson 1982; Rumsey and Wilson 1996; Wilson and Vinogradov 2014, 2015). The rate of oxygen reduction represents a net forward rate (v_O_) through reactions (5) and (6) represented by subtracting v_O_ from the reverse rate of each reaction. As the concentration of glucose increases, the input of reducing equivalents from glucose needs to be added to v_R_ and the ratio becomes:

(8) $$V_{F}/\left(V_{R}-V_{O}+V_{B}\right)$$

**Figure 3:**
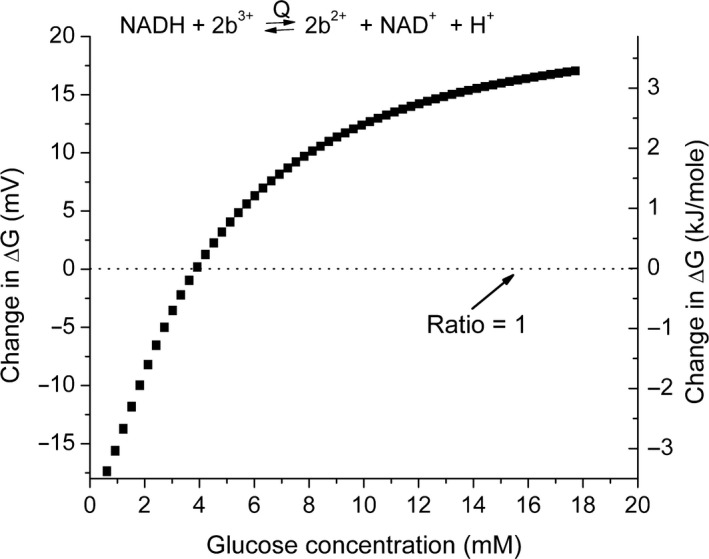
Displacement of reaction 6 from equilibrium and its dependence on glucose concentration. In the absence of glucose, there is a net flow of reducing equivalents through reaction B for oxygen reduction. The G‐6‐P produced by glucokinase introduces flow of reducing equivalents into the cytochrome b region of the respiratory chain and, through pyruvate dehydrogenase, into the citric acid cycle. The effect on reaction 6 is calculated from the model and the predicted displacement from equilibrium presented as both the change in oxidation‐reduction potential across the coupling site in mV and the ΔG in kJ/mole. The zero crossing point is where the input of reducing equivalents from glucose matches the output for oxygen reduction.

**Figure 4:**
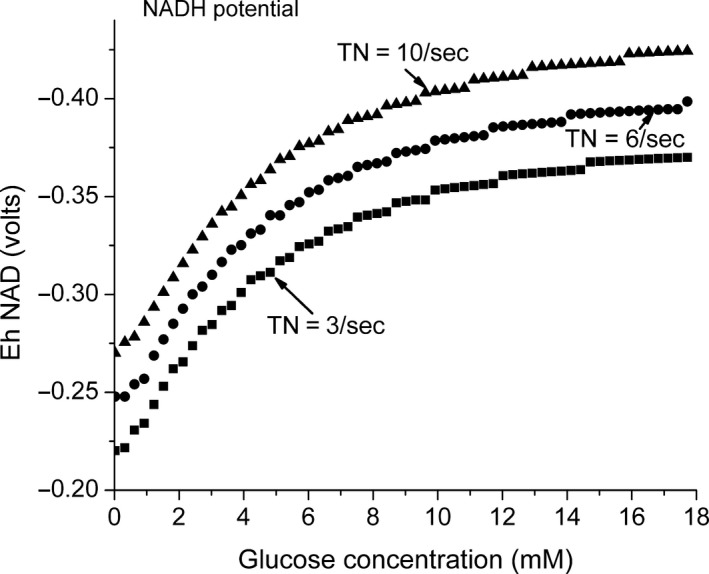
The predicted oxidation–reduction potential for the intramitochondrial NAD couple as a function of glucose concentration. The input of reducing equivalents from glucose increases the potential difference across the coupling sites of oxidative phosphorylation through reaction B. This increase is distributed across the three coupling sites. The predicted changes in redox potential of the NAD couple for cytochrome c turnover numbers of 3, 6, and 10 s^−1^ (equivalent to oxygen consumption rates of 6, 12, and 20 *μ*mol/L s^−1^) are presented in the graph.

The model makes use of equations (7) and (8). The ratio v_F_/v_R_ was calculated from the equilibrium constant and then the rate constants increased proportionally until v_F_/(v_R_ − v_O_) is close to 1 (reaction is near equilibrium). For reaction 5, the rates are high enough that input from glucose does not significantly alter the ratio. For reaction 6, however, the rates are lower and the input from glucose is higher, resulting a glucose concentration–dependent change in the calculated ratio. For the model, the values for the forward and reverse rates for reaction 6 were increased until v_F_/(v_R_ − v_O_) was approximately 1.3 for the initial conditions (no glucose). When glucose is added, v_B_ is added to the reverse reaction, v_F_/(v_R_ − v_O_ + v_B_), the change in the ratio, expressed as the change in ΔG, can be calculated as a function of glucose concentration. This is shown in Figure 3 where the calculated change in ΔG, expressed in mV and kJ/mole, is plotted against glucose concentration. In the absence of glucose, the net flux is in the forward direction and the calculated ΔG is negative. As the glucose concentration increases, the input of reducing equivalents reaches and then exceeds that used for oxygen reduction and the net flux reverses (calculated ΔG becomes positive). The result of the influx of reducing equivalents from glucose is progressive reduction of the intramitochondrial NAD couple (Fig. 4). As the electrochemical potential of the NAD couple becomes more negative, the energy coupled to ATP synthesis, and therefore to the energy state, also increases. The change in potential of the NAD couple as a function of glucose concentration is substantial and responsible for the change in energy state.

The level of reduction of the intramitochondrial NAD couple is difficult to measure experimentally, so there are insufficient data for good comparison with the predictions of the model. Trus et al. (1980) reported that, for isolated perifused rat islets, increased glucose resulted in an increase in total NADH. Total amount includes both the mitochondrial and cytoplasmic compartments and does not distinguish between the NADH that is protein bound and that which is free. The increased reduction of the NAD couple predicted by the model is, in contrast, for the free concentrations in the intramitochondrial compartment. As a result, the experimental observation that the total NADH in islets increased is encouraging but not very strong support for the model. The decrease in the intramitochondrial [NAD^+^]/[NADH] predicted by the model is large, however, and should be observed in metabolite couples associated with intramitochondrial dehydrogenases. The levels of glutamate are reported to increase (Schuit et al. 1997), which may result in part from increased NAD reduction acting through glutamate dehydrogenase.

### Steady‐state predictions of the model compared to experimental measurements

**Figure 5:**
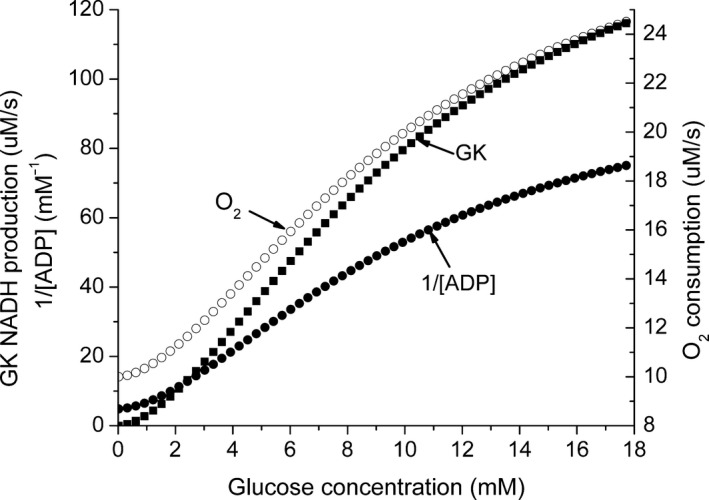
The dependence of the predicted rates of glucokinase and oxygen consumption as well as of 1/[ADP] on glucose concentration. The model uses the kinetic expression for the glucokinase reaction to calculate the rate of G‐6‐P production. The rate of oxygen consumption is calculated following the experimental observation that it increases about twofold with increasing glucose concentration and the increase is proportional to the rate of G‐6‐P production. Oxygen consumption in the absence of glucose is assumed to be equivalent to a cytochrome c TN of 4 s^−1^. The predicted 1/[ADP] is included to show that the curve is very similar to those for GK activity and O_2_ consumption.

**Figure 6:**
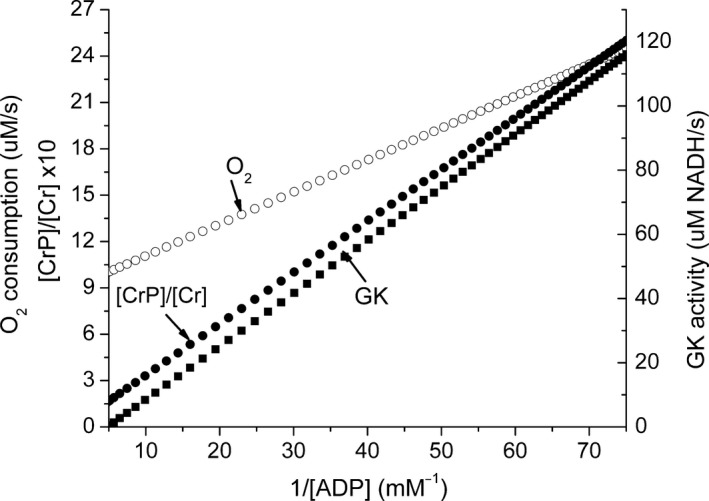
The predicted correlation of GK activity and O_2_ consumption with 1/[ADP]. The GK and O_2_ consumption rates are plotted against 1/[ADP], showing that the model predicts a strictly linear correlation of both GK activity and O_2_ consumption with 1/[ADP]. The [CrP]/[Cr] ratio is also plotted and this is, as expected, linearly correlated with 1/[ADP].

The rate of oxygen consumption is determined by the rate of ATP utilization, and there is no a priori reason for the experimentally observed increase in oxygen consumption with increase in glucose concentration. The intracellular Na^+^ concentration has been reported to decrease (Wesslen et al. 1986; Doliba et al. 2003), suggesting that increased ion transport is at least partially responsible for the increase in ATP consumption. The experimental data show that the rate of oxygen consumption and the energy state both increase with increasing glucose concentration (Liang 1996; Doliba et al. 2003, 2012; Sweet et al. 2005). At first glance, this seems odd because the respiratory rate is usually observed to increase when the energy state decreases and to decrease when energy state increases. The experimental data show that the increase in oxygen consumption is nearly proportional to the rate of glucose metabolism, increasing about twofold as the glucose concentration increases from 2 to 24 mmol/L. A similar increase in oxygen consumption is incorporated into the model by making v_O_ proportional to v_G_ such that at high (20 mmol/L) glucose concentration v_O_ increases to approximately twice the value at 1 mmol/L glucose. Figure 5 shows the predicted rates of the glucokinase reaction and oxygen consumption plotted as a function of glucose concentration. The dependence of 1/[ADP] on glucose concentration is also shown, and the shape of the curve is very similar to those for the GK reaction and oxygen consumption. When the rates of the GK reaction and oxygen consumption are plotted against 1/[ADP] (Fig. 6), there is a linear correlation between reciprocal ADP and both oxygen consumption and G‐6‐P production. The [CrP]/[Cr] ratio predicted by the model is also included in Figure 6, and it is also linearly correlated with 1/[ADP].

### The effect of increasing glucose concentration on the cellular energy state

**Figure 7:**
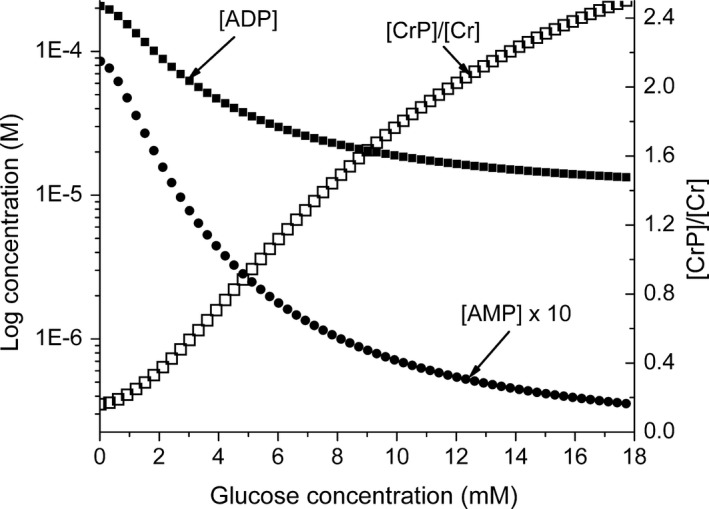
The dependence of ADP, AMP, and [CrP]/[Cr] on glucose concentration. One of the most interesting and important questions in glucose sensing is the effect of GK activity on the energy state and in particular ADP. The prediction of the model, shown in the figure, is for the ADP to decrease from about 200 *μ*mol/L at zero glucose to about 13 *μ*mol/L as the glucose increases to 18 mmol/L. The decrease in AMP is much larger, from near 10^−5^ mol/L to about 3 × 10^−8^ mol/L. The [CrP]/[Cr] ratio is included to show the increase in [ATP]/[ADP] associated with the decrease in ADP. The increase in [CrP]/[Cr] is from about 0.2 to more than 2.

As noted earlier, the increased reduction of the intramitochondrial NAD pool increases the energy available for ATP synthesis. The predicted dependence of the [CrP]/[Cr] ratio and the concentrations of ADP and AMP on glucose concentration are shown in Figure 7. As the glucose concentration increases, there is a substantial decrease in ADP and increase in [CrP]/[Cr]. As glucose increases from 1 to 18 mmol/L the, ADP falls by about 80%, from near 120 *μ*mol/L to less than 15 *μ*mol/L. In most cells, the adenylate kinase reaction is near equilibrium, so AMP decreases much more than does ADP, from near 4 *μ*mol/L to about 0.04 *μ*mol/L. The [CrP]/[Cr] ratio increases from about 0.25 to 2.5. In vivo there are additional fuel sources, notably amino acids, and these prevent the energy state from falling to pathological levels in hypoglycemia. This is included in the model as a basal rate of NADH production equivalent to a cytochrome c turnover near 4s^−1^. Experimental measurements using isolated islets (Matschinsky and Ellerman 1968; Trus et al. 1980; Ghosh et al. 1991; Wroblewski et al. 1994; Doliba et al. 2003) show the total concentrations of both ADP and AMP decrease with increase in glucose concentration, suggesting that the free concentrations also decreased. The available experimental measurements are total concentrations, however, whereas the model predictions are for the free concentrations, so the absolute numbers are not readily compared. The predicted creatine phosphate/creatine ratios are consistent with those observed experimentally (Ghosh et al. 1991; Ronner et al. 2001; Doliba et al. 2003).

**Figure 8:**
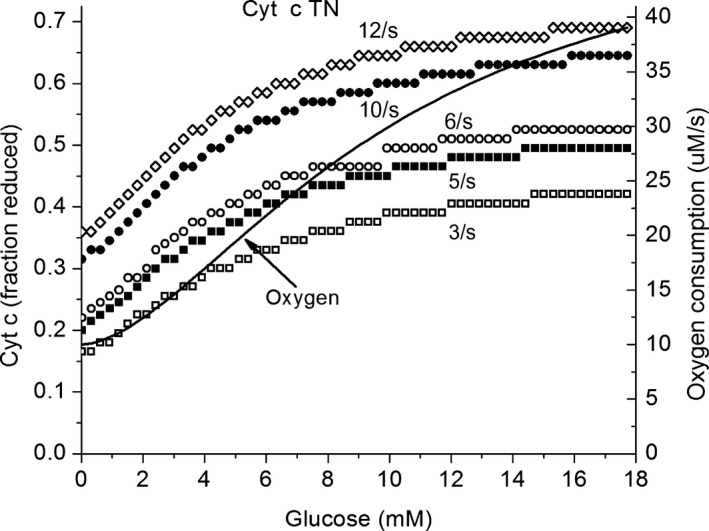
The effect of increasing GK activity on the state of reduction of cytochrome c. Cytochrome c in the respiratory chain has been measured in perifused rat islets, and there is a marked reduction with increased glucose concentration (Sweet et al. 2005). In the figure, the predicted fraction of cytochrome c reduced is presented for cytochrome c turnover numbers of 3, 5, 6, 10, and 12 s^−1^. In each case, cytochrome c reduction increases markedly with increase in glucose concentration. The increase in oxygen consumption with increase in glucose concentration is also plotted (line) in order to show how that change in rate affects the level of cytochrome c reduction.

It has been reported that, in perifused islets, increasing glucose concentrations are associated with large increases in the level of reduction of cytochrome c (Sweet et al. 2005). The predicted effect of glucose concentration on cytochrome c reduction is shown in Figure 8. The calculations have been made for different rates of oxygen consumption expressed as the turnover number for cytochrome c. The concentration of cytochrome c in *β*‐cells is assumed to be 8 *μ*mol/L and perifused islets to have a volume of 2 nL. The resulting turnover numbers range from 4 s^−1^ at low glucose concentration to about 12 s^−1^ at 20 mmol/L glucose. The effect of increasing glucose concentration on the level of reduction of cytochrome c, as predicted by the model, has been calculated for cytochrome c turnover numbers of 3, 5, 6, 10, and 12 s^−1^. These turnovers are equivalent to oxygen consumption rates in islets of 6, 10, 12, 18, 20, and 24 *μ*mol/L O_2_ s^−1^. The curves are for individual rates of respiration, whereas in islets the rate of oxygen consumption increases. A curve for the respiratory rate with the glucose concentration is also presented in the figure, and where this line intercepts the curves for constant cytochrome c, TN shows the predicted level of reduction for isolated islets.

Sweet et al. (2005) reported cytochrome c reduction increased from 29% to 68%, when the glucose concentration was increased from 3 to 20 mmol/L. This is in good agreement with the increase from 25% to 70% predicted by the model for similar conditions. It should be noted that the level of reduction of cytochrome c is dependent on the rate of ATP utilization, energy state, and oxygen pressure. Sweet et al. (2005) did not have a way to measure the energy state for the experiments in which the cytochrome c reduction was measured. In addition, the perfusate oxygen pressure was held constant and increased extraction of oxygen from the perfusate would have increased the diffusion gradient and decreased the oxygen pressures inside the islets. How much the decrease would be and how much it might have contributed to the reduction of cytochrome c remain unknown. That the model predicts, and experiment shows, increased reduction of cytochrome c and that the increases are similar is encouraging.

## Discussion

**Figure 9:**
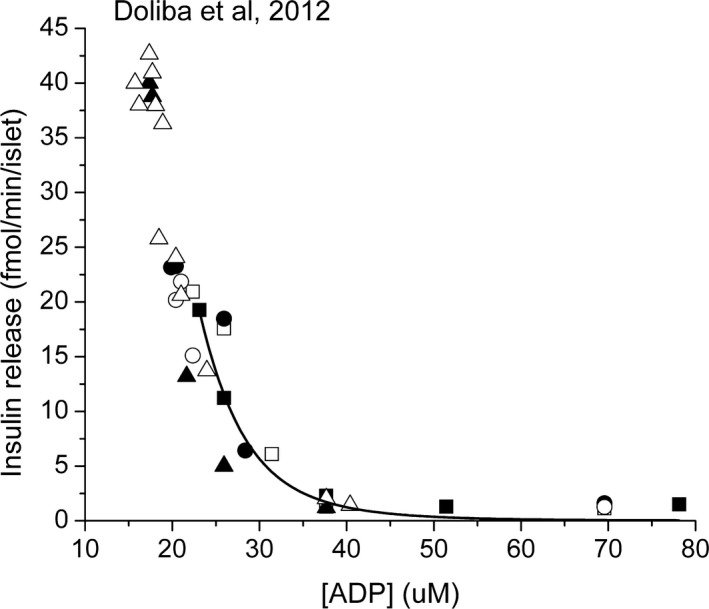
Correlation of the release of insulin from perifused islets and the concentration of ADP predicted by the model. Doliba et al. (2012) measured the rate of insulin by islets from rats, mice and humans, reporting that all three showed the same correlation between insulin release and the rate of synthesis of ATP calculated by combining ATP synthesis by glycolysis and oxidative phosphorylation. The model was used to calculate the ADP for each rate of ATP synthesis and the insulin release data (raw data courtesy of Dr. Doliba) plotted against ADP. The curve is sigmoidal and best fit to the Hill equation (Origin) has a threshold near 30 *μ*mol/L ADP, a maximal release rate of 65 fmol/min/islet and a negative Hill coefficient near −5.5.

In order for *β*‐cell metabolism to function as a robust glucose sensor, it is necessary that the utilization of fuels other than glucose, such as fatty acids and amino acids, be the minimum needed for cell survival in the absence of glucose. In addition, their rate of utilization should change little or decrease with increasing glucose concentration. Doliba et al. (2012) reported that islets isolated from rats, mice and humans all show the same correlation between release of insulin and increase in the rate of ATP synthesis. When the data were plotted with insulin release on the ordinate and ATP synthesis rate on the abscissa, they were well fitted by a sigmoidal curve with a Hill coefficient of 11 and a threshold for insulin secretion at 15 pmole ATP/min/islet (125 *μ*mol/L s^−1^). Interestingly, the model predicts a linear relationship between the rate of ATP synthesis and 1/[ADP]. This allows the ADP at each rate of ATP synthesis, as predicted by the model, to be plotted against release of insulin (Fig. 9). The threshold for increasing insulin release with decreasing ADP is about 30 *μ*mol/L, whether calculated by extrapolation of the region of linear decrease in insulin release or simply observing that significant increase in insulin release occurs when the ADP falls to about 30 *μ*mol/L. This level of ADP is consistent with measurements in *β*‐cells (Ghosh et al. 1991; Ronner et al. 2001; Doliba et al. 2003). The dependence on ADP is sigmoidal and can be reasonably fitted by the Hill equation with a negative Hill coefficient near −5.5, and a Vm near 60 fmol insulin/min/islet. The measurements and model predictions are consistent with inhibition of insulin release by high ADP and a highly cooperative mechanism for insulin release that is turned on as ADP decreases. The value of the threshold ADP is a function of ATP and Pi, becoming smaller with decreasing ATP and with increasing Pi. For the present calculations, the ATP and Pi were assumed to be 5 mmol/L and 6 mmol/L, respectively. The energy state at the threshold for insulin release is the same as homeostatic set point that is programmed in oxidative phosphorylation (Wilson et al. 1979; Wilson and Vinogradov 2014, 2015; Wilson 2015a, 2015b, 2016, 2017). It is important to note that AMP concentrations change more than those of ADP, and AMP would appear to be a good messenger for control of insulin release. It would not be surprising if AMP concentration has a role in glucose sensing and AMP‐dependent regulatory mechanisms, such as AMP‐dependent protein kinase (Zhou et al. 2001; Rutter 2003; Ruderman and Prentki 2004; Zang et al. 2004; Lamontagne et al. 2009), participate in glucose sensing.

Support for our minimal model is further strengthened by the observation that genetic mutations in glucokinase and in the K‐ATP channel are responsible for defects in glucose homeostasis in humans (Froguel et al. 1993; Otonkoski et al. 2003, 2007; Ashcroft 2005; Osbak et al. 2009). These studies clearly established the primary roles of glucokinase and the K‐ATP/SUR‐1 potassium channel complex in the signaling pathway that couples blood glucose levels to the release of insulin (Fig. 1). Mutations in the genes of either part of the signaling pathway that result in altered activity result in diabetic (hypoinsulinemic) or hypoglycemic (hyperinsulinemic) phenotypes depending on whether the mutation results in activation or inhibition. These striking observations on the genetic basis of diabetes and hypoglycemia demonstrate how important glucokinase and the K^+^ channel are to maintaining the normal, 5–7 mmol/L, range of blood glucose in mice, rats, and humans (Matschinsky 1990; Ashcroft 2005; Osbak et al. 2009).

Although glucokinase is responsible for sensing the glucose concentration, the information generated from G‐6‐P by glycolysis must be specifically and faithfully transmitted to oxidative phosphorylation to achieve an appropriately effect on the energy state. This transmission depends on the activity of the monocarboxylate carrier and of lactic dehydrogenase being much lower than that of glucokinase. If a large fraction of the pyruvate were converted to lactate and exported or exported as pyruvate, this would “short circuit” signal transmission. Similarly, restriction of the glycerol phosphate shuttle should limit glycolysis and glucose sensing, resulting in hyperglycemia. Mugabo et al. (2016) reported that there is a glycerolphosphate phosphatase in mammalian cells that hydrolyzes glycerol phosphate to glycerol. This enzyme was shown to play a significant role in regulating metabolism in hepatocytes and *β*‐cells, possibly through modulation of the cellular concentrations of glycerol phosphate and thereby the activity of the shuttle. Schuit et al. (1997) reported that in purified *β*‐cells minimal lactate was produced from glucose but high pyruvate carboxylase activity resulted in production of oxalacetate (anaplerosis) and carbon from glucose accumulated as glutamate and malate as well as in newly synthesized protein. Acetyl‐CoA is required for pyruvate carboxylase activity but the affinity for acetyl‐CoA increases with increasing pyruvate (Scrutton 1974), resulting in the activity increasing with increase in pyruvate even if the concentration of acetyl‐CoA does not change. The dependence of the activity on pyruvate is sigmoidal with a Hill coefficient near 2, so the activity of pyruvate carboxylase increases with increasing pyruvate through both increase in its substrate concentration and increased affinity for acetyl‐CoA. Elevated pyruvate concentrations also activate pyruvate dehydrogenase phosphatase (McCormack et al. 1990; Holness and Sugden 2003), which activates PDH. Thus, the fraction of the pyruvate oxidized (f) is a balance between the rates of lactate production, pyruvate carboxylation, and PDH activity (about 20%, 40%, and 20%) with each of the rates being regulated by the level of pyruvate. Achieving the right balance in the distribution of pyruvate is critical to glucose sensing. Alteration in the activity of pyruvate carboxylase in *β*‐cells would alter glucose sensing, increase causing hyperglycemia, and decrease hypoglycemia, other parameters being unchanged. Increase or decrease in lactate and pyruvate transport activity in *β*‐cells is reported (Ricordi et al. 1990; Otonkoski et al. 2007) to cause hyper‐ and hypo‐glycemia, respectively. Increased transport activity is also reported to cause exercise‐induced hyperinsulinemia (Otonkoski et al. 2003), in which blood lactate and pyruvate levels increase and these are taken up by the *β*‐cells. This lowers the glucose concentration required for release of insulin and induces hypoglycemia. Changes in PDH or pyruvate carboxylase activities, if localized to the *β*‐cells, would also interfere with glucose sensing, but these enzymes are essential to metabolism in most cells and widespread pathology would mask the effects on the *β*‐cells.

One aspect of glucose sensing that is implied by the model and observed in the experimental data is that at high glucose concentrations the production of NADH and of carbon‐containing products substantially exceeds the amounts accounted for by respiration and CO_2_ production. For NADH, the reducing equivalents produced reach 150% of those used to reduce oxygen and only about 40% of the carbon in the glucose appears as lactate and CO_2_. This leaves 60% of the carbon that is being turned into other metabolites. The identity and quantity of the “missing” products is important to the chemical balance (output must be equal to input) and to understanding metabolism in *β*‐cells. Production of metabolites such as glutamate, aspartate, and malate from pyruvate through carboxylation by pyruvate carboxylase (Schuit et al. 1997), combined with the lactate production and oxidation of pyruvate through PDH, can reasonably account for at least 80% of the reducing equivalents and carbon from the metabolized glucose. It is important to note that these “extra” pathways for carbon from G‐6‐P are essential to glucose sensing. The input from glucose is by an irreversible reaction and the output has to equal input or excessive accumulation of the intermediates of glycolysis will occur. The extra pathways have two important functions: (1) to provide a “safety valve” that prevents excessive accumulation of the intermediates should the downstream reactions be slowed for any reason, and (2) to increase the response time for the regulation, i.e. when glucose and the level of intermediates is high and glucose decreases, these “leak” reactions facilitate decrease in the levels of the intermediates. In the absence of the extra pathways for carbon removal, the control system would be both “brittle” in that it would be ultra sensitive to any and all of the core reactions and have a slower response time.

The role of oxidative phosphorylation in glucose‐dependent release of insulin through control of K^+^ channel conductance is augmented by signaling pathways such as the AMP‐dependent protein kinase (AMPK), which add an additional level of regulation (Zhou et al. 2001; Ruderman and Prentki 2004; Zang et al. 2004; Lamontagne et al. 2009; Madiraju et al. 2014). Activation of AMPK is reported to suppress, and inhibition to enhance, insulin release. The current model does not address this additional level of regulation, focusing only with the core metabolism in the sensing system.

### In summary

A mechanism‐based model for glucose sensing has been presented and the predictions of the model shown to be consistent with most of the data reported in the literature. Glucokinase is responsible for the initial response to (measurement of) glucose concentration while oxidative phosphorylation is responsible for the changes in the cellular energy state that trigger depolarization and release of insulin. The activity of glucokinase is coupled to oxidative phosphorylation by a combination of the NADH oxidation through the glycerol phosphate shuttle and pyruvate oxidation through PDH and the citric acid cycle. The model integrates the metabolic parts and provides an integrated picture of glucose sensing by *β*‐cells. This includes a quantitative expression describing the interactions among the metabolic parameters. The fit of the quantitative expression to the available experimental data provides credence for the mechanisms incorporated into the model. It also indicates that the model has significant predictive power, and carrying out virtual experiments using the model can greatly improve experimental design and data collection as well as aiding in interpretating the obtained data.

## Conflict of Interest

None declared.

## Data Accessibility
